# Implementation of Systematic Childbirth Education Through a Birth Planning Visit at a Tertiary Care Hospital in India: A Quality Improvement Project

**DOI:** 10.7759/cureus.111787

**Published:** 2026-06-30

**Authors:** Shivangi Mangal, K Aparna Sharma, Piyush Ranjan, Rinchen Zangmo, Deepali Garg, Vatsla Dadhwal

**Affiliations:** 1 Obstetrics and Gynecology, All India Institute of Medical Sciences, New Delhi, New Delhi, IND; 2 Medicine, All India Institute of Medical Sciences, New Delhi, New Delhi, IND; 3 Obstetrics and Gynecology, Ysbyty Gwynedd, Bangor, GBR

**Keywords:** birth companion, birth plan counseling, birth plans, information on contraception, labor analgesia, prenatal exercises, prenatal or antenatal education, quality improvement project

## Abstract

Background and aim

Antenatal education and birth planning are essential components of respectful maternity care, improving childbirth experiences by preparing families for labor. Knowledge about prenatal exercises, birth companions, pain relief, delivery methods, and postpartum contraception empowers couples during childbirth and enhances the quality of care. Despite evidence supporting routine birth plans, our antenatal clinics lacked a formal policy for prenatal education. This study aimed to establish the practice of birth plan counseling from an existing rate of 0% and increase it to 50% within 10 months using the Point of Care Quality Improvement methodology.

Methods

Following baseline data collection and a fishbone analysis of existing deficits, five sequential Plan-Do-Study-Act cycles were implemented: (1) physician-led counseling and stakeholder training; (2) integration of paramedics and physiotherapists via video consultations; (3) transition of services to ultrasound rooms during COVID-19 outpatient closures; (4) implementation of trimester-wise room redistribution for group counseling; and (5) transfer of responsibilities to permanent family planning staff to ensure sustainability after resident graduation.

Results

Over the 10-month period, birth plan clinic attendance increased from a median of 0% to 50% and was sustained for an additional six months. Secondary outcomes also improved, including postpartum contraception adoption (from 28% to 54.25%), prenatal exercise participation (from 10% to 57%), and labor analgesia use (from 0% to 50%), although birth companion presence decreased from 40% to 29% because of pandemic-related social distancing mandates.

Conclusions

Systematic healthcare worker training and methodical staff utilization successfully established antenatal counseling practices. Implementing new healthcare practices requires a multistep approach with system-level changes, while routine monitoring ensures sustainability.

## Introduction

Every woman has a right to respectful maternity care, which includes the provision of information, obtaining informed consent, and honoring choices that strengthen her capability to give birth [1]. Antenatal education aims to positively influence health behaviors and build confidence, while structured birth plans outline patients’ preferences during labor and the postpartum period. Evidence demonstrates that women who receive comprehensive antenatal education experience less fear, anxiety, and stress while gaining increased childbirth self-efficacy, higher vaginal birth rates, and improved trust and communication with their healthcare providers [2,3]. Furthermore, structured counseling sessions regarding postpartum contraception provided during birth planning significantly increase acceptance and continuation rates among couples, effectively reducing short birth intervals [4-6]. Conversely, the acceptance of prenatal exercises remains low in regions lacking established institutional counseling programs [7].

Comprehensive birth plans should educate women regarding labor processes, pain relief options, potential complications, and breastfeeding policies while actively emphasizing prenatal exercises and the presence of a labor companion [8-11]. In alignment with these goals, the LaQshya program, launched by the Ministry of Health and Family Welfare, Government of India, is a national labor room quality improvement (QI) initiative that explicitly emphasizes the critical role of the birth companion [12].

While implementation literature remains limited in this context, the WHO’s Point of Care Quality Improvement (POCQI) framework offers a validated methodology for introducing such systemic changes [13]. Effective counseling ultimately empowers women, alleviates maternal anxiety, and encourages preparation for vaginal delivery, resulting in higher patient satisfaction regardless of strict adherence to the initial plan [14-17].

At our institute, no formal prenatal education or birth planning was conducted, and limited healthcare staff were involved in promoting physical activity and prenatal exercises. Thus, this QI project was implemented with the aim of establishing the practice of birth plan formation, including the five essential components of antenatal counseling (i.e., antenatal exercises, birth companion, mode of delivery, labor analgesia, and postpartum contraception) by using the principles of QI, which included analysis of the problem and implementation of Plan-Do-Study-Act (PDSA) cycles as a step-by-step approach to provide quality care.

To the best of our knowledge, no study has been conducted in India on the implementation of a routine birth planning visit to provide antenatal education for a positive childbirth experience.

This article was previously posted to the Authorea preprint server on August 21, 2025.

## Materials and methods

This study was conducted in the Department of Obstetrics and Gynecology at the All India Institute of Medical Sciences, New Delhi, from January 2021 to October 2021, with a six-month sustainability phase extending to May 2022, following the Standards for Quality Improvement Reporting Excellence (SQuIRE) 2.0 guidelines. Ethical approval was obtained from the Institute Ethics Committee (approval no. IECPG-320/22.07.2020, RT-43/26.08.2020).

**Figure 1:**
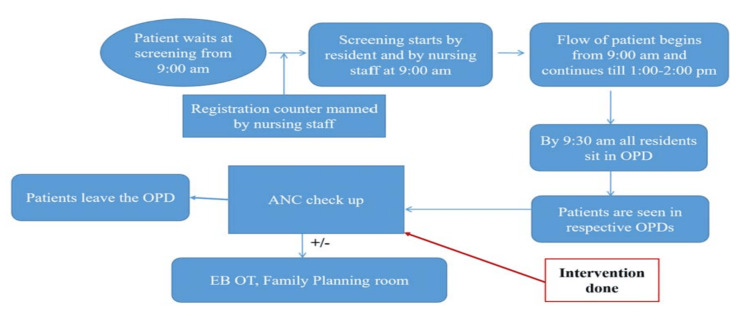
Process flow map ANC, antenatal care; EB OT, emergency obstetric operation theater

**Figure 2:**
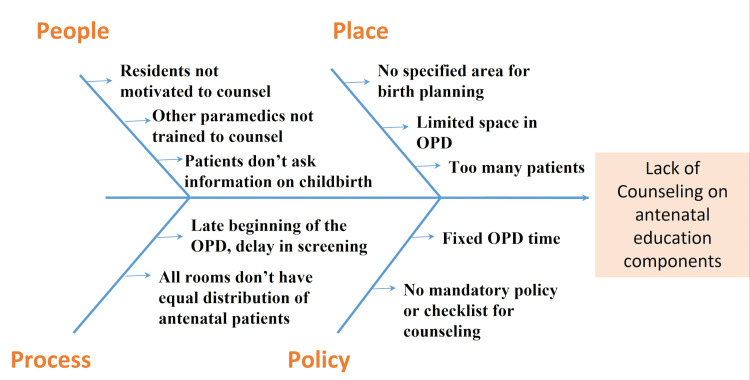
Ishikawa (fishbone) analysis

As a tertiary referral center serving high-risk pregnancies primarily from low- to middle-socioeconomic backgrounds, the department runs antenatal clinics three times per week but lacked formal birth plan counseling at baseline. Consecutive sampling was used to enroll all pregnant women presenting during the third trimester (32-34 weeks of gestation), excluding those requiring immediate inpatient admission. A multidisciplinary QI team was formed, led by a junior resident and comprising faculty members, nursing officers, family planning counselors, and physiotherapists. The team used a baseline process flow map and an Ishikawa (fishbone) analysis to identify key gaps in personnel, space, policy, and processes (Figure 1, Figure 2).

The primary objective was to establish a structured, routine third-trimester birth plan counseling program for antenatal women covering the five essential components of birth planning (i.e., antenatal exercises, birth companion, mode of delivery, labor analgesia, and postpartum contraception). The secondary objective was to evaluate the sustainability of these measures over a subsequent six-month maintenance phase after the targets had been achieved. The primary outcome was the percentage of women enrolled in the birth planning visit among the total number of women who delivered during the study period.

A SMART aim is one that is specific, measurable, achievable, relevant, and time-bound, transforming a general goal into a measurable target. In this study, the SMART aim (primary outcome) was to increase the proportion of booked antenatal patients who attended a third-trimester birth planning visit that included counseling on the five components in the antenatal OPD from a baseline of 0% to 50% over the next 10 months (January 2021 to October 2021).

Primary and secondary outcomes were recorded using an OPD pro forma, compiled in Microsoft Excel (Microsoft Corporation, Redmond, WA, USA), and monitored biweekly using run charts. Outcomes included the percentages of total delivered patients enrolled in birth planning visits, prenatal exercise participation (excluding women with medical contraindications), birth companion presence during labor, labor analgesia use, and postpartum contraception uptake. Five sequential PDSA cycles were implemented at eight-week intervals. The target median for prenatal exercise participation, labor analgesia acceptance, and postpartum contraception acceptance was 50%, compared with baseline values of 10%, 0%, and 28%, respectively. The target median for birth companion presence during labor was 75%, compared with a baseline of 40% (Table 1).

**Table 1:** Defined outcome indicators

Outcome indicator	Definition
Primary outcome	Percentage of women enrolled in the birth planning visit among the total number of women who delivered during the study period.
Secondary outcome: Prenatal exercises accepted	Percentage of eligible women enrolled in antenatal exercises by trained educators among those enrolled during the birth planning visits. Women who were not eligible for enrollment were excluded from the denominator for this outcome indicator.
Secondary outcome: Birth companion present during labor	Percentage of eligible women who delivered in the presence of a birth companion among those enrolled during the birth planning visits.
Secondary outcome: Labor analgesia accepted	Percentage of eligible women who opted for labor analgesia (pharmacological or nonpharmacological) among those enrolled during the birth planning visits.
Secondary outcome: Postpartum contraception	Percentage of eligible women who adopted postpartum contraception among those enrolled during the birth planning visits.

All low-risk antenatal women in their third trimester who presented to the OPD were recruited for prenatal counseling and birth planning. Antenatal women with high-risk pregnancies (placenta previa, preeclampsia with severe features, antepartum hemorrhage, uncontrolled hypertension or diabetes mellitus, threatened preterm labor, or a history of recurrent pregnancy loss) and women presenting to the emergency department who were admitted directly to the inpatient department were excluded from birth plan counseling. A sample size calculation was not performed because all eligible women visiting the OPD were included in the study. None of the women declined to participate.

Baseline data collection through OPD observation over two weeks revealed that no women received formal birth plan education in any OPD room. Counseling gaps were identified across all five key areas. Run charts were plotted fortnightly using a standardized template. Shifts in median values indicated significant system changes. Because the denominators varied over time, the data are presented as percentages only.

In PDSA cycle 1, the team developed several change ideas to address the lack of birth plan counseling. Standardized infographics in the form of wall posters were created covering prenatal exercises, birth companionship, modes of delivery, labor analgesia, and postpartum contraception options, including PPIUCD, injectable progesterone, barrier contraception, oral contraceptive pills, and tubal ligation. The counseling content was standardized according to the WHO recommendations for a positive pregnancy experience. Checklists were developed that included the counseling components, and the resident completed the checklist after each counseling session. A designated counseling room was established where junior residents provided one-on-one counseling sessions (15-20 minutes) for women at 32-34 weeks’ gestation. Residents and faculty were encouraged to refer eligible patients for counseling after their routine appointments. Following counseling, the women’s antenatal cards were stamped to indicate that they had attended the session. To facilitate data tracking, a birth planning visit column was added to the antenatal ward register.

After eight weeks, the median percentage of women who attended the birth planning clinic among the total number of booked antenatal women increased from 0% to only 9%. The reasons for not achieving the target outcome were analyzed. Two main issues emerged: physicians often forgot to refer antenatal women for counseling, and approximately 75% of counseled women missed their physiotherapist appointments because of scheduling conflicts and extended waiting times for the physiotherapist in the OPD.

For PDSA cycle 2, the change ideas from PDSA cycle 1 were adapted. Nursing officers were encouraged to actively refer women from the OPD screening area to the counseling room. Faculty and residents received training and reinforcement on counseling. Physiotherapists were asked to teach women about prenatal exercises via videoconferencing, enabling patients to schedule appointments outside regular OPD hours. Physiotherapists also counseled women online if they lacked sufficient time during their hospital visit. WhatsApp video calls (Meta Platforms, Inc., Menlo Park, CA, USA) and Google Meet sessions (Google LLC, Mountain View, CA, USA), which had been introduced during PDSA cycle 1, were continued.

The interventions implemented during PDSA cycle 2 improved the median outcome of prenatal education to 42% over the next eight weeks. Before the implementation of the next PDSA cycle and further planned interventions, the COVID-19 pandemic forced the closure of routine OPDs during April-June 2021. The counseling process continued via telephone consultations. During the OPD closure, counseling was provided by the resident in charge using WhatsApp and Google Meet, following the same standardized content and checklists used for in-person counseling. Because the same resident conducted all counseling sessions, the quality and content of counseling remained consistent. However, the median percentage of women attending the birth planning clinic decreased to 35.8%. This necessitated adaptations to address pandemic-related challenges.

In PDSA cycle 3, following the closure of OPD rooms, counseling was shifted to the ultrasound rooms, where women at 32 weeks’ gestation attended growth scans. Birth companionship was difficult during the COVID-19 pandemic because of social distancing requirements and measures to prevent the spread of infection; therefore, it was temporarily discontinued in the labor rooms. One-on-one counseling was provided by the resident in the ultrasound rooms. Physiotherapists provided in-person counseling on prenatal exercises, while video calls were conducted for women who could not attend in person. After each physiotherapy session, the physiotherapists sent confirmation of the counseling to the resident. No further improvement in the outcomes was observed over the next eight weeks. The busy schedule of the resident, the high volume of antenatal women in the ultrasound rooms, and the lack of seating and a designated counseling area were identified as major contributing factors. Patients were also dissatisfied with the increased waiting time. Therefore, these changes were abandoned, and another PDSA cycle was planned.

In PDSA cycle 4, after the COVID-19 pandemic ended, routine OPDs resumed. The change idea was to redistribute the OPD rooms to ensure that all women in their third trimester were referred directly to the counseling room, with referrals coordinated by the nursing officers in the OPD screening area. A second change idea involved counseling women in small groups (a maximum of six women per session) to increase attendance at the birth planning clinic. The median increased to 44% over the next eight weeks. These changes were further strengthened and adopted.

**Figure 3:**
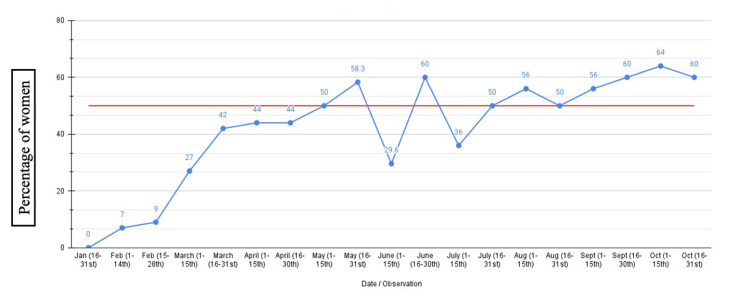
Run chart showing the median outcome after five PDSA cycles Blue: outcome indicator (%) measured every two weeks; red line: median of the outcome indicator; X-axis: two-weekly data points; Y-axis: percentage of women who received antenatal counseling among the total number of women booked for delivery during the corresponding time period PDSA, Plan-Do-Study-Act

In PDSA cycle 5, routine antenatal OPDs continued. A designated family planning room was assigned for birth plan counseling, a register was created for data collection, and family planning staff were trained to counsel women. OPD paramedical staff directed patients to the counseling room after their consultations. For one month, the QI resident oversaw counseling across all three clinic days. Antenatal cards were stamped after counseling, and physiotherapists were assigned a separate room directly opposite the counseling room for prenatal exercise training. Fixed days and times helped establish prenatal education and birth planning as a routine part of antenatal visits. The median increased from 44% to 50% within eight weeks, achieving the SMART aim and enabling transition to the sustainability phase (Figure 3).

**Figure 4:**
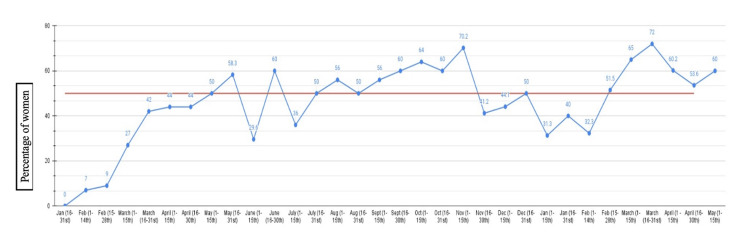
Run chart of outcomes during the sustainability phase Blue: outcome indicator (%) measured every two weeks; red line: median of the outcome indicator; X-axis: two-weekly data points; Y-axis: percentage of women who received antenatal counseling among the total number of women booked for delivery during the corresponding time period

The six-month sustainability phase focused on hardwiring the implemented changes. Family planning staff received biweekly reinforcement to maintain the counseling process. Senior faculty and residents conducted fortnightly visits during counseling hours to ensure that the content remained adequate, accurate, and complete. During the sustainability phase (Figure 4), we initially observed a decrease in the percentage of women receiving birth plan counseling. This decline resulted from difficulties in directing antenatal women in the OPD to the designated birth planning room. Family planning staff found it time-consuming to direct patients to the birth planning room. To address this challenge, we collaborated with OPD paramedical staff and security guards to establish a direct patient flow from the entry gate to the birth planning room. Patients were directed to the birth planning room immediately after their routine checkup.

From December 2021 to February 2022, the percentage of women who attended the birth planning visit remained low because the third COVID-19 wave affected OPD attendance and staff availability through increased sick leave, although the median was maintained. Despite these challenges, the family planning staff consistently provided birth plan counseling.

The outcome indicator improved again from February 2022 as the COVID-19 wave subsided. The median remained at 50% during the six-month sustainability phase (November 2021 to May 2022), demonstrating the sustainability of the QI initiative.

## Results

Following the sequential implementation of five PDSA cycles over a 10-month period, the primary outcome indicator, the proportion of booked third-trimester antenatal women who attended the birth planning clinic among the total number of women who delivered, demonstrated a significant and sustained system shift, increasing from a baseline of 0% to a post-intervention median of 50%. This target was maintained throughout the six-month sustainability phase (Table 2).

**Table 2:** Summary of PDSA cycles and results PDSA, Plan-Do-Study-Act; SMART, specific, measurable, achievable, relevant, and time-bound

Cycle	Plan	Do	Study	Act
PDSA cycle 1 (January 20, 2021 to March 10, 2021)	(A) Develop infographics. (B) Designate a location and personnel for counseling. (C) Develop a counseling checklist and stamp.	(A) Five posters were created. (B) A designated birth planning area was established. (C) One-on-one counseling was provided on the five key components. (D) Residents and faculty were encouraged to refer eligible patients.	(A) Median increased to 9%. (B) Not all women were referred. (C) Three-fourths of counseled women did not attend their physiotherapy appointments.	Adapt the changes.
PDSA cycle 2 (March 11, 2021 to May 15, 2021)	(A) Physiotherapists agreed to provide their contact numbers to patients. (B) Retraining and reinforcement of residents.	(A) Orientation was conducted by the resident in the birth planning room.	(A) Median increased to 42% but later declined to 35.8% because of the COVID-19 pandemic.	Adapt the changes to address challenges during the COVID-19 pandemic.
PDSA cycle 3 (May 15, 2021 to July 7, 2021)	(A) Counsel women during ultrasound visits. (B) Temporarily discontinue birth companionship because of the COVID-19 pandemic.	(A) Counseling was conducted by the resident in the ultrasound room.	(A) Median reached 39%. (B) Barriers identified included limited resident time to provide counseling while performing ultrasound examinations. (C) Patient waiting times increased.	Abandon the changes.
PDSA cycle 4 (July 8, 2021 to August 31, 2021)	(A) Redistribute OPD rooms. (B) Implement group counseling.	(A) Group counseling was conducted following the revised OPD room distribution.	(A) Median increased to 44%.	Adopt the changes.
PDSA cycle 5 (September 2021 to October 2021)	(A) Expand counseling across all units. (B) Train family planning staff and residents. (C) Introduce new stamps. (D) Establish a counseling register. (E) Designate a family planning room.	(A) Counseling was expanded. (B) Counseling days and times were standardized.	(A) Median increased to 50%. (B) SMART aim achieved.	(A) Continue reinforcement. (B) Adopt the practice.

The run chart after five PDSA cycles showed six consecutive data points above the median line, indicating a clear median shift according to the Institute for Healthcare Improvement run chart interpretation standards. This demonstrated that the target had been achieved and that the outcome had improved significantly from baseline. At the end of the sustainability phase, the primary outcome remained at the target level, demonstrating the sustainability of the QI initiative.

**Figure 5:**
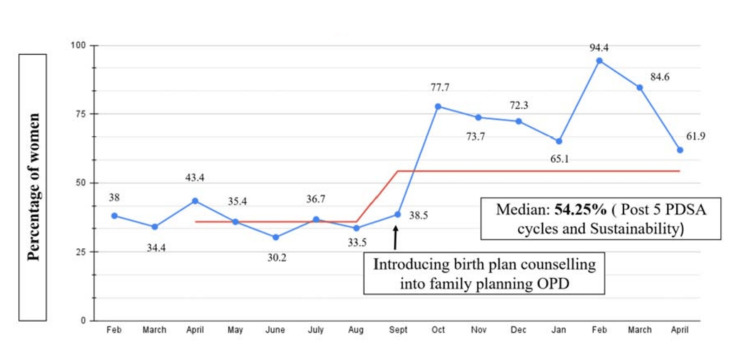
Run chart of the outcome after contraceptive counseling Red line: median of the outcome indicator; blue line: monthly outcomes; X-axis: timeline; Y-axis: percentage of women accepting a contraceptive method among those attending the birth planning clinic PDSA, Plan-Do-Study-Act

**Figure 6:**
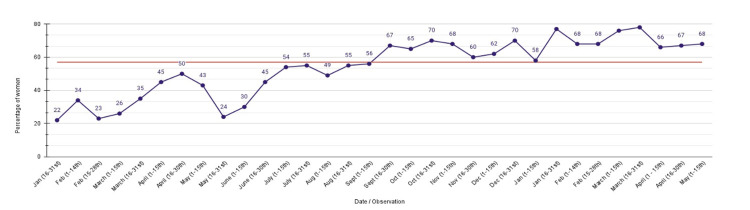
Two-weekly run chart of prenatal exercise participation among antenatal women Red line: median of the outcome indicator; blue line: two-weekly outcomes; X-axis: timeline; Y-axis: percentage of women participating in prenatal exercises among those attending the birth planning clinic

**Figure 7:**
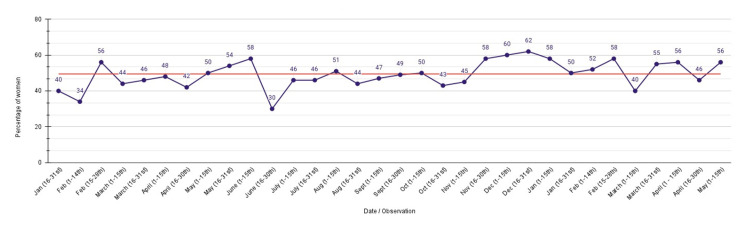
Two-weekly run chart of labor analgesia acceptance Red line: median of the outcome indicator; blue line: two-weekly outcomes; X-axis: timeline; Y-axis: percentage of women accepting a method of labor analgesia among those attending the birth planning clinic

**Figure 8:**
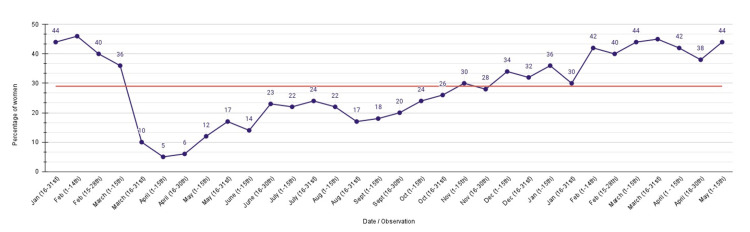
Two-weekly run chart of birth companion presence during delivery Red line: median of the outcome indicator; blue line: two-weekly outcomes; X-axis: timeline; Y-axis: percentage of women who had a birth companion during delivery among those attending the birth planning clinic

Marked improvements were also observed across the secondary clinical outcomes among women who received structured counseling (Table 3). The median rate of postpartum contraception adoption increased from a baseline of 28% to 38% after the initial PDSA cycles and ultimately reached 54.25% by the end of the sustainability phase (Figure 5). Similarly, participation in structured prenatal exercises increased from a baseline of 10% to 45% after the intervention (Figure 6) before stabilizing at 57% during the sustainability phase. Patient acceptance and use of labor analgesia (pharmacological or nonpharmacological) increased from a baseline median of 0% to 46% and ultimately reached the target median of 50% during the sustainability phase (Figure 7). Conversely, because of institutional social distancing policies and infection control measures implemented during successive waves of the COVID-19 pandemic, the proportion of deliveries attended by a birth companion declined from a baseline of 40% to 22% and recovered only slightly to 29% by the end of the study, falling short of the secondary SMART target of 75% (Figure 8).

**Table 3:** Summary of secondary outcome indicators PDSA, Plan-Do-Study-Act; SMART, specific, measurable, achievable, relevant, and time-bound

Component	Baseline	SMART aim	After five PDSA cycles	Sustainability phase	Target achieved
Postpartum contraception acceptance (Figure 5)	28%	50%	38%	54.25%	Yes
Prenatal exercise participation (Figure 6)	10%	50%	45%	57%	Yes
Labor analgesia acceptance (Figure 7)	0%	50%	46%	50%	Yes
Birth companion present during labor (Figure 8)	40%	75%	22%	29%	No

Chronologically, PDSA cycle 1 established standardized infographics, a dedicated counseling room, and an antenatal card-stamping system, increasing the median to 9%; however, clinicians frequently missed referrals, and 75% of women encountered scheduling barriers for physiotherapy appointments. PDSA cycle 2 addressed these issues by training nursing staff in the screening area to actively refer eligible women and by introducing virtual physiotherapy consultations, increasing the median to 42% before COVID-19 restrictions necessitated temporary teleconsultations, reducing the median to 35.8%. During PDSA cycle 3, one-on-one counseling was relocated to the ultrasound rooms during routine growth scans because of OPD closures. However, this strategy was abandoned because resident time constraints and prolonged patient waiting times reduced its feasibility. In PDSA cycle 4, following the resumption of routine OPD services, third-trimester patients were redistributed to facilitate small-group counseling, increasing the median to 44%. Finally, PDSA cycle 5 institutionalized the intervention by transferring counseling responsibilities to family planning staff, establishing dedicated registers, standardizing clinic schedules, and locating the physiotherapy room adjacent to the counseling room, thereby achieving the SMART target of 50%.

During the six-month sustainability phase, initial challenges with patient routing were addressed by collaborating with OPD paramedical staff and security personnel to establish a direct patient flow from the clinic entrance to the birth planning room. Despite temporary staffing shortages and increased sick leave during a subsequent COVID-19 wave between December 2021 and February 2022, the intervention remained sustainable, and the median coverage of birth planning counseling was maintained at 50%.

## Discussion

This QI initiative demonstrated that structured birth plan counseling can be effectively integrated into routine antenatal care through systematic, low-cost interventions guided by the POCQI methodology. The project targeted improvements in five key components of childbirth counseling and established a formal counseling pathway for antenatal women. Over five PDSA cycles, birth planning visits increased from 0% to 50%, with sustained outcomes over a six-month follow-up period, confirming both feasibility and sustainability.

A critical observation was the limited impact of personnel-dependent strategies, such as sensitization of residents, which failed to produce sustained improvements. In contrast, system-level interventions, including direct channeling of third-trimester women, group counseling, visual aids, and task shifting to trained family planning staff, resulted in significant and sustained gains. The incorporation of paramedical staff and the use of technology further enhanced service delivery and patient engagement.

These findings are consistent with the existing literature emphasizing iterative adaptation and the feasibility of interventions. In 2025, Thakur et al. demonstrated that initial counseling strategies were insufficient to improve expressed breast milk use; however, subsequent system-based modifications, including ward-based counseling, telephonic reminders, and protocol standardization, increased early breast milk initiation from 38.7% to 100% [18]. Similarly, in 2018, Dudeja et al. highlighted the importance of feasibility in intervention design, showing that initiating breastfeeding in the operating theater, rather than the recovery room, improved rates from 0% to 93% [19]. Sharma et al. reported that simple policy restructuring reduced waiting times for infertility treatment from six to 3.25 months, underscoring the impact of process redesign [20]. Furthermore, Maria et al. achieved an increase in skin-to-skin contact from 0% to 100% through policy changes and procedural innovations, while Sharma et al. demonstrated substantial improvements in early and exclusive breastfeeding through task redistribution to nursing students [21,22]. This emphasizes the role of systematic changes rather than personnel-level changes, which help produce the desired outcomes and sustain them despite personnel rotation. It is worth noting that, although secondary outcomes such as contraception uptake and prenatal exercise participation improved under this system redesign, the proportion of deliveries attended by a birth companion paradoxically decreased because of strict institutional social distancing mandates and infection control policies enforced during the height of the COVID-19 pandemic.

Several challenges were encountered during the PDSA cycles, including high patient volume, dependence on residents for referrals, missed referrals, limited physiotherapy visits, and disruption of OPD services during the COVID-19 pandemic. These were addressed through direct channeling from the screening area, group counseling, telephone- and video-based physiotherapy support, trimester-wise room allocation, staff training, and supervision. The experience highlights that sustainable improvement requires system-based changes rather than person-dependent interventions.

Additionally, evidence from a systematic review by Rowe et al. indicates that combining training with supervision yields greater improvements in healthcare practices than training alone [23]. Collectively, these findings reinforce that sustainable QI outcomes are driven by system redesign, iterative testing, and optimal utilization of available human resources rather than reliance on individual effort alone.

The study had a few limitations. It did not evaluate the effects of prenatal exercises, birth companion presence, or labor analgesia on mode of delivery or maternal and neonatal outcomes. Additional limitations include the lack of a control group for direct comparison of the outcomes of birth planning counseling. This QI initiative aimed to enroll all eligible women for birth planning counseling; therefore, the study lacked a fixed denominator because the number of bookings fluctuated over time. The study also did not assess the knowledge, attitudes, and practices of counseled women. Furthermore, the generalizability of this QI initiative beyond a high-resource tertiary care center may be limited because of resource and infrastructure constraints in lower-resource settings where large numbers of women deliver. Another limitation is the potential for selection bias because only low-risk women who were able to attend birth planning counseling were included; therefore, the findings may be inherently skewed toward stable, lower-risk outpatients. Lastly, no formal audit was conducted to assess the quality or consistency of counseling provided by physiotherapists.

## Conclusions

The present study demonstrates that integrating comprehensive antenatal education through structured birth planning visits is both feasible and operationally sustainable in a high-volume tertiary care setting. Birth planning improved the acceptance of labor analgesia, prenatal exercises, and postpartum contraception. The target for birth companion practice could not be achieved because of system-level disruptions during the COVID-19 pandemic. The successful establishment of this routine counseling pathway was achieved not by increasing institutional financial costs but through the systematic utilization of existing human resources combined with structured training, iterative staff reinforcement, and a methodical, multistep QI framework. Providing structured counseling empowers expectant mothers by increasing awareness of their reproductive rights and choices, thereby enhancing maternal autonomy, reducing childbirth-related anxiety, and promoting a positive childbirth experience. Moving forward, continuous quality monitoring and institutional oversight remain essential to preserve counseling fidelity and long-term adherence. Future research should focus on optimizing healthcare worker training models, objectively evaluating longitudinal knowledge retention among pregnant women, and assessing the direct impact of specific birth planning components, such as active birthing positions and birth companion integration, on maternal and neonatal clinical outcomes, as well as overall maternal childbirth satisfaction.
